# Phthisiophobia

**Published:** 1902-02-01

**Authors:** 


### The Hospital

A Journal of

The Medical Sciences and Hospital Administration.

Vol. XXXI.—No. 801.] Edited by Sir Henry Burdett, K.C.B., and Solomon C. Smith, M.D., M.R.C.P. [February 1, 1902.

### PHTHISIOPHOBIA.

England is often reproached with being a slow-going, torpid and conservative country, and although this reproach may be in some senses true, these characteristics at least confer upon us the advantage of being able to profit by the mistakes of our more go-ahead neighbours. The particular mistake to which we would on the present occasion draw attention is that of attributing too great and too exclusive an importance to infection in the production of tuberculosis, and our reason for reverting to it at the present moment is that there is to be seen on every side an exaggerated and a growing dread of having anything to do with those who suffer from consumption.

People at large decline to draw fine distinctions, and however many sides there may be to a question they much prefer to see only one at a time. Hence many difficulties, and it is to be feared some cruelties. Sanitarians, bent on teaching people the importance of taking measures to avoid dust, darkness, dried sputum, and overcrowding, have freely used the word "infection" in regard to tuberculosis as a handy and effective sledge-hammer by which to drive their moral home. But those to whom all this teaching has been addressed have cared but little for details; the word "infection" was quite enough for them; they jumped to what they would call the natural conclusion that consumptive patients are infectious, and that the less they have to do with them the better; and it is because this tendency to ostracise, to turn adrift, and to refuse all association with those who are suspected of being consumptive is rapidly growing in our midst, and is likely to entail terrible hardships upon a class of people who are already hit hard enough by the disease, that we would point to what has lately happened in America as a warning. In the United States this view of the contagiousness of the consumptive has been carried to its really logical conclusion; pulmonary tuberculosis has been declared to be a dangerous contagious disease, and an order has been issued debarring all immigrants affected with it from entering the country. There is no doubt that in so acting the officials responsible for this proceeding have received the approbation of a considerable section of the American public—approbation which must be taken for what it is worth—but at a recent meeting of the New York Academy of Medicine where the whole subject was discussed by physicians who saw the real bearing of this new departure, the evil consequences which must follow the acceptance of such a false and erroneous view of the tuberculosis problem were strongly insisted upon. Dr. Knopf, a well-known authority on the subject of tuberculosis, pointed out what is true enough, that the acceptance of the idea that consumption is a "dangerous contagious disease" cannot end with the mere exclusion of a few more or less helpless immigrants, but must affect the ordinary daily movements of the "thousands of American citizens" suffering from tuberculosis. That is where the rub comes; if we are to accept this theory of the infectivity of the consumptive, rather than of the disease—a theory which is daily exercising more and more influence—it at once becomes a question not of isolating a few helpless and far-gone consumptives, but of interfering with the daily life, the incoming and the outgoing of the working and bread-winning sufferers from tuberculosis; an enormous task, for, according to a recent statement by Dr. Vivian Poore, this disease affects in one way or another one-seventh of the population. Dr. Knopf showed that the effect of the recent action of the immigration authorities, supported as it has been by the courts, has been to give official sanction to that growing and exaggerated fear of the vicinity of consumptives to which he gives the name of phthisiophobia. He told the meeting that under the influence of this fear even healthy employees have been discharged because some one of their near relatives with whom they were living had been reported to be suffering from consumption, and he mentioned case after case in which people had lost their situations under the influence of this growing dread of all that is related to consumption. "How much suffering and hardship may thus be daily created only those," he says, "who come in contact with poor consumptives can know." In America theories are quickly acted upon. Not only has American public opinion already excluded consumptive immigrants, but it is now demanding that tuberculous travellers shall have special railway carriages set apart for them. Indeed, some go so far as to maintain that the phthisiophobia against which Dr. Knopf declaimed is to be encouraged, since it tends "to force the tuberculous cases out of the crowded tenement districts," and drive them into hospitals. The last instance of this phthisiophobia, of which we have heard, is directed against the hospitals themselves, being a resolution of the Health Board of a district in the State of New York, prohibiting the maintenance of any hospital or sanatorium for consumptives within the area under their jurisdiction. All this harrying of the consumptives is a terrible mistake, and must lead to monstrous cruelty. Let us then in dealing with this disease stick to what is proven, namely, that it is the conditions in which the people live which give the bacillus its opportunity, that it is the dried sputum that carries the "infection," and that as regards the patient himself, if he takes proper care nothing can be farther from the mark than the recent pronouncement of the American authorities that he is suffering from a "dangerous contagious disease."

